# Artificial neuronal network analysis in investigating the relationship between oxidative stress and endoplasmic reticulum stress to address blocked vessels in cardiovascular disease

**DOI:** 10.5937/jomb0-33855

**Published:** 2022-10-15

**Authors:** Fatma Kalay, Toprak Muhammet Sait, Hakan Ekmekçi, Mine Kucur, Barış İkitimur, Hüseyin Sönmez, Zeynep Güngör

**Affiliations:** 1 University of Istanbul - Cerrahpasa, Cerrahpasa Medical School, Department of Medical Biochemistry, Istanbul, Turkey; 2 University of Istanbul - Cerrahpasa, Cerrahpasa Medical School, Department of Cardiology, Istanbul, Turkey

**Keywords:** artificial neural network analysis, atherosclerosis, cardiovascular disease, endoplasmic reticulum stress, oxidative stress, analiza veštačke neuronske mreže, ateroskleroza, kardiovaskularne bolesti, stres endoplazmatskog retikuluma, oksidativni stres

## Abstract

**Background:**

Cardiovascular disease is the leading cause of death in the world and is associated with significant morbidity. Atherosclerosis is the main cause of cardiovascular disease (CVD), including myocardial infarction (MI), heart failure, and stroke. The mechanism of atherosclerosis has not been well investigated in different aspects, such as the relationship between oxidative stress and endothelial function. This project aims to investigate whether an oxidative enzyme vascular peroxidase 1 (VPO1) and activating transcription factor 4 (ATF4) can be used as biomarkers in highlighting the pathogenesis of the disease and in evaluating the prognosis of the relationship with endoplasmic reticulum and oxidative stress. This paper used artificial neural network analysis to predict cardiovascular disease risk based on new generation biochemical markers that combine vascular inflammation, oxidative and endoplasmic reticulum stress.

**Methods:**

For this purpose, 80 patients were evaluated according to the coronary angiography results. hs-CRP, lipid parameters and demographic characteristics, VPO1, ATF4 and Glutathione peroxidase 1(GPx1) levels were measured.

**Results:**

We found an increase in VPO1 and hs-CRP levels in single-vessel disease as compared to controls. On the contrary, ATF4 and GPx1 levels were decreased in the same group, which was not significant. Our results showed a significant positive correlation between ATF4 and lipid parameters. A statistically significant positive correlation was also observed for VPO1 and ATF4 (r=0.367, P<0.05), and a negative correlation was found for ATF4 and GPx1 (r=-0.467, P<0.01). A significant negative relationship was noted for GPx1 and hs-CRP in two/three-vessel disease (r=-0.366, P<0.05). Artificial neural network analysis stated that body mass index (BMI) and smoking history information give us an important clue as compared to age, gender and alcohol consumption parameters when predicting the number of blocked vessels.

**Conclusions:**

VPO1 and ATF4 might be potential biomarkers associated with coronary artery disease, especially in the follow-up and monitoring of treatment protocols, in addition to traditional risk factors.

## Abstract (sr)

**Uvod:**

Kardiovaskularne bolesti su vode}i uzrok smrti u svetu i povezane su sa značajnim morbiditetom. Ateroskleroza je glavni uzrok kardiovaskularnih bolesti (CVD), uključujući infarkt miokarda (MI), srčanu insuficijenciju i moždani udar. Mehanizam ateroskleroze nije dobro istražen u različitim aspektima, kao što je odnos izmenu oksidativnog stresa i funkcije endotela. Ovaj projekat ima za cilj da istraži da li se oksidativni enzim vaskularna peroksidaza 1 (VPO1) i aktivirajući transkripcioni faktor 4 (ATF4) mogu koristiti kao biomarkeri koji bi ukazali na patogenezu bolesti, kao i u proceni prognoze veze sa stresom endoplazmatskog retikuluma i oksidativnim stresom. Ovaj rad je koristio analizu veštačke neuronske mreže za predviđanje rizika od kardiovaskularnih bolesti na osnovu biohemijskih markera nove generacije koji kombinuju vaskularnu inflamaciju, oksidativni stres, kao i stres endoplazmatskog retikuluma.

**Metode:**

U tu svrhu, posmatrano je 80 pacijenata prema rezultatima koronarne angiografije. Mereni su hs-CRP, parametri lipida i demografske karakteristike, nivoi VPO1, ATF4 i glutation peroksidaze 1 (GPk1).

**Rezultati:**

Pronašli smo povećanje nivoa VPO1 i hs-CRP kod bolesti jednog suda u poređenju sa kontrolnom grupom. S druge strane, nivoi ATF4 i GPk1 su smanjeni u istoj grupi, što nije bilo značajno. Naši rezultati su pokazali značajnu pozitivnu korelaciju između parametara ATF4 i lipida. Statisti~ki zna~ajna pozitivna korelacija uočena je i za VPO1 i ATF4 (r=0,367, P<0,05), a negativna za ATF4 i GPk1 (r=-0,467, P<0,01). Značajna negativna veza je zabeležena za GPk1 i hs-CRP kod bolesti sa dva ili tri krvna suda (r=-0,366, P<0,05). Analiza veštačke neuronske mreže je pokazala da nam indeks telesne mase (BMI) i podaci o istoriji pušenja daju važan trag u poređenju sa parametrima starosti, pola i konzumiranja alkohola kada se predviđa broj blokiranih krvnih sudova.

**Zaključak:**

Pored tradicionalnih faktora rizika, VPO1 i ATF4 mogu biti potencijalni biomarkeri povezani sa bole{}u koronarnih arterija, posebno u praćenju i nadgledanju protokola lečenja.

## Introduction

Endothelial dysfunction is an early stage of many cardiovascular diseases, such as atherosclerosis, coronary heart disease, and hypertension. Oxidative stress promotes endothelial dysfunction via several mechanisms such as increasing lipid, protein oxidation, or inflammatory burden.

Oxidized low-density lipoproteins (ox-LDLs) contribute to the formation and progression of atherosclerotic lesions by promoting local inflammation and toxic events, which might cause endothelial dysfunction [1] [2]. The subendothelial retention of low-density lipoprotein (LDL) and its oxidative form, oxidized LDL (ox-LDL), activates signalling pathways resulting in an oxidative stress response, which leads to atherosclerosis [3] [4].

Oxidative stress plays a crucial role in abdominal aortic aneurysm (AAA) formation and regulates VSMC phenotypic switch. Hydrogen peroxide (H_2_O_2_), an important key reactive oxygen species, acts as an important signalling molecule that modulates VSMCs [5].

A heme-containing peroxidase, called VPO1 (vascular peroxidase 1), is mainly expressed by vascular endothelial cells, cardiac, and smooth muscle cells [6]. VPO1 plays a critical signalling role in mediating the development and progression of cardiovascular disease [7] [8] [9]. It generates hypochlorous acid (HOCl), which is a strong reactive oxygen substance (ROS) from hydrogen peroxide (H_2_O_2_) and chloride anion (Cl). Recently, it was also reported that HOCl contributes to the acceleration of endothelial ageing in hyperlipidemic rats or in ox-LDL-treated cells [10].

The main player of the energy metabolism, mitochondria, contributes to ROS formation via high energy metabolism. While ROS are vital for life, due to their high chemical reactivity, growing evidence support that some organelles such as ER need ROS to appreciate their function. The ER is responsible for correcting misfolding of proteins into their functional conformations. The disulfide bonds play a crucial role during this process which is assisted by protein disulfide isomerases (PDI), endoplasmic reticulum oxidoreductin 1 (ERO1), and glutathione (GSH) [10].

Of the machine learning techniques used in predicting coronary artery disease, neural network analyses are popularly used to improve performance accuracy. Neural network analyses make it possible to discover new patterns and information related to coronary artery disease by analyzing complex data [11].

We, therefore, hypothesized that the changes of VPO1 activity during the progression of atherosclerosis might affect ER stress which induces antioxidant gene expression (ATF6). To test this hypothesis, we investigated VPO1 activity, ATF6 and hs-CRP levels, and glutathione peroxidase-1 activity according to the coronary angiography results in patients with cardiovascular disease.

## Materials and methods

A total of 80 individuals, 20 patients with N-vessel disease, 30 patients with one vessel disease, 30 patients with two-three vessel disease from Cerrahpasa Medical School, Department of Cardiology, at the University of Istanbul-Cerrahpasa, were enrolled in this study. The study protocol was previously reviewed and approved by the Ethics Committee of the University of Istanbul, Cerrahpasa Medical School (Issue Number: 83045809-604.01.02, 03.05.2016). Written informed consent was obtained from all participants. All blood samples were collected following the Declaration of Helsinki.

Patients with chronic liver disease, chronic renal failure, cancer, serious systemic infections, chronic lung disease, or any endocrine disease were excluded. The patients were angiographically determined. According to their angiography results, subjects were split into three groups as defined above. The patients with no-vessel disease (N vessel disease) had no obstructed vessels. Still, they suffered from chest pain similar to angina pectoris. All of them were admitted with unstable angina pectoris. None of them were significant alcohol consumers.

### Samples

Samples for the determinations of vascular peroxidase 1 (VPO1), transcription activation factor 4 (ATF4), glutathione peroxidase 1 (Gpx1), hs-C-reactive protein (hs-CRP), and lipid parameters were obtained from venous blood after a 12 h fasting by centrifugation of clotted specimen within 30 min. It was centrifuged at 4400 rpm for 10 min. The separated serum and plasma samples were stored in several small aliquots at − 30°C until being assayed.

Total cholesterol (TC), high density lipoprotein (HDL), low density lipoprotein (LDL), triglyceride (TG) and hs-CRP levels were analyzed in the autoanalyzer of the faculty's central biochemistry laboratory.

Enzyme-linked immunosorbent assay (ELISA) procedure was used to determine the serum VPO1 (Human Peroxidasin Homolog (PXDN)-(CUSABIO, CSB-EL019106HU-USA)), ATF4-transcription activation factor-4 (MYBIOSOURCE-MBS762729-USA) and Gpx1 (CUSABIO, CSB-EL009866HU-USA), levels according to the procedure from the manufacturer. CRP was measured immunoturbidimetrically in routine clinical chemistry analyzer (Roche, Hitachi, Basel, Switzerland) by using reagents purchased from the same manufacturer.

### Statistical analysis

Analysis of data was done with SPSS statistical analysis software (version 20.0; SPSS Inc, Chicago,IL, USA). Results were expressed as means ± SD or median and interquartile range. ATF4, Gpx1, hs-CRP levels were logarithmically transformed to achieve normal distributions. Correlation analysis was done by Pearson correlation analysis. All analyses were twotailed, and P-values less than 0.05 were considered statistically significant. Then we applied artificial neural network analysis to check the importance of our parameters on blocked vessels. In the analysis; age, gender, CAD history, smoking and alcohol comsumption defined as a factor. BMI, VPO1, ATF4, Gpx1, hs-CRP, HDL, LDL, total cholesterol, triglyceride defined as covariates.

## Results

Clinical characteristics of 80 patients according to the angiographic results were summarized in Table 1. Mean age of the patients was similar (55, 59 and 60, respectively; P>0.05). There were no significant differences between BMI and the lipid parameters among groups (P>0.05).

**Table 1:** Clinical characteristics of the study population. Data are presented as means ±SD or as median (interquartile range: 25–75%) for non-normally distributed variables. P values were calculated using ANOVA, and values for triglyceride levels were logarithmically transformed before analysis. Non-transformed values are shown. NS: not significant, significance level: P<0.05

	N Vessel Disease<br>N=20	One Vessel Disease<br>N=30	Two-three Vessel Disease<br>N=30	P-Value
Age	55±9	59±8	60±8	0.054
BMI, kg/m^2^	28.4±3.9	29.2±4.2	29.7±4.6	NS
Triglyceride, mmol/L	1.706 (0.915–2.994)	1.841 (1.378–2.508)	1.548 (0.983–2.293)	NS
Total Cholesterol, mmol/L	5.3617±1.450	4.895±1.372	5.335±1.191	NS
LDL-Cholesterol, mmol/L	3.626±1.217	3.237±1.191	3.703±0.984	NS
HDL-Cholesterol, mmol/L	1.243±0.259	1.061±0.336	1.113±0.310	NS

ATF4, VPO1, Gpx1 and hs-CRP levels in patients according to the blocked vessels were shown in Table 2. The decline in ATF4 level was observed in one vessel and two-three vessel disease as compared to N-vessel disease. A significant decrease was detected in one vessel disease compared to N-vessel disease (1.57 vs 1.96; P< 0.05). No significant change was seen for other parameters among groups (P>0.05). We recognized a decline in Gpx1 levels in one vessel and two/three-vessel disease as compared to N-vessel disease (114, 117 vs 143, respectively), but this difference did not reach significance (P>0.05).

**Table 2:** ATF4, VPO1, Gpx1 and hs-CRP levels in patients with N vessel, one vessel and two-three vessel disease. Data are presented as means±SD or as median (interquartile range: 25–75 %) for non-normally distributed variables. P values were calculated using ANOVA, and values for ATF4, Gpx-1andhs-CRP levels were logarithmically transformed before analysis. Non-transformedvalues are shown. NS: not significant, significance level: P<0.05. Benferroni test was used for binary comparison ^*^; represents a comparison of ATF4 levels between no vessel disease and one vessel disease.

	N-Vessel Disease<br>N=20	One-Vessel Disease<br>N=30	Two-three Vessel Disease<br>N=30	P-Value
VPO1, ng/mL	5.97±2.58	6.11±2.93	5.33±2.20	NS
ATF4, ng/mL	1.96 (1.69–2.54)	1.57 (1.05–1.92)^*^	1.74 (1.46–2.10)	0.043
Gpx1, μU/L	143.14 (85.28–248.96)	114.65 (57.12–145.02)	117.33(70.37–146.31)	NS
Hs-CRP, mg/L	2.14 (0.37–4.51)	2.33 (0.88–7.38)	3.50 (1.25–7.46)	NS

**Figure 1:**
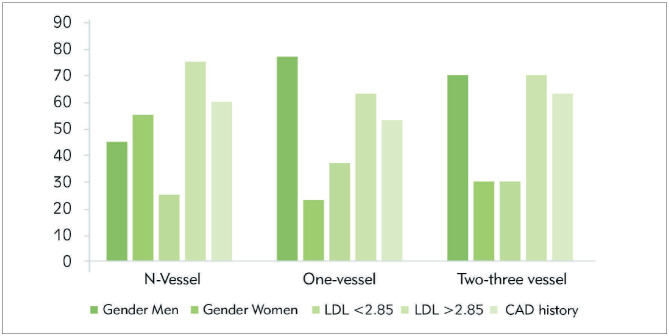
Proportion of critical values of the hospital during 2019 (before optimization) and 2020 (after optimization).

The percentage of blocked vessel disease increases among men (45%, 77%, 70%, respectively) as compared to women counterpart (55%, 23%, 30%, respectively). The prevalence of LDL highness (75%, 63.3%, 70%, respectively), and CAD history (60%, 53.3%, 19%, 63.3%) were for N-vessel, one vessel, two/three vessel disease, respectively (Figure 1).

Afterward, we checked associations of the biochemical markers within the subjects. A significant but opposite relationship was observed between VPO1 and Gpx1 (r=-0.285, P<0.05). No significant association was seen for other parameters in Table 3. We next analyzed the correlations between the same biochemical and lipid parameters in Table 4. A significant, positive association were observed between ATF4 and triglyceride (r=0.294, P<0.001), total cholesterol (r=0.359, P<0.001), and LDL cholesterol (r=0.317, P<0.001). No other significant correlations were observed between other counterparts.

**Table 3:** Correlations between biochemical parameters. Values for ATF4, Gpx1, and hs-CRP levels were logarithmically transformed before analysis. ^*^ P<0.05

	VOP1	ATF4	GPx1	hs-CRP
VPO1		0.175	-0.285^*^	-0.161
ATF4	0.175		0.041	-0.089
Gpx1	-0.285^*^	0.041		-0.065
hs-CRP	-0.161	-0.089	-0.065	

**Table 4:** Correlations between lipid parameters and VPO1, ATF4, Gpx-1, and hs-CRP. Values for ATF4, Gpx1 and hs-CRP levels were logarithmically transformed before analysis. ^**^P<0.01

	Triglyceride	Total<br>Cholesterol	LDL<br>Cholesterol	HDL<br>Cholesterol
VPO1	0.104	0.144	0.120	0.156
ATF4	0.294^**^	0.359^**^	0.317^**^	-0.012
Gpx1	-0.057	-0.026	0.009	0.077
hs-CRP	0.011	0.053	0.080	-0.086

Regarding the observed relationship, which was seen in Table 3 and Table 4, we next checked the similar correlations among the same biochemical parameters across blocked vessel disease. We could not find any significant association for N-vessel disease. We found a positive and significant association between VPO1 and ATF4 (r=0.367, P<0.05), also a negative and significant association between ATF4 and Gpx1 (r=-0.467, P<0.01). In two-three vessel disease counterpart, we observed a negative but significant correlation between hs-CRP and Gpx1 levels (r=-0.366, P<0.05) (Table 5).

**Table 5:** Correlations between biochemical parameters among N-vessel, one, two-three vessel disease. Values for ATF4, Gpx-1andhs-CRP levels were logarithmically transformed before analysis. ^*^P<0.05 ^**^ P<0.01

	VPO1	ATF4	Gpx-1	hs-CRP
N Vessel Disease				
VPO1		0.042	-0.273	0.236
ATF4	0.042		-0.266	0.076
Gpx-1	-0.273	-0.266		-0.097
hs-CRP	0.236	0.076	-0.097	
One Vessel Disease				
VPO1		0.367^*^	-0.467^**^	-0.297
ATF4	0.367^*^		-0.022	-0.216
Gpx-1	-0.467^**^	-0.022		0.063
hs-CRP	-0.297	-0.216	0.063	
Two-three Vessel Disease				
VPO1		0.149	0.194	-0.281
ATF4	0.149		0.134	-0.074
Gpx-1	0.194	0.134		-0.366^*^
hs-CRP	-0.281	-0.074	-0.366^*^	

**Figure 2:**
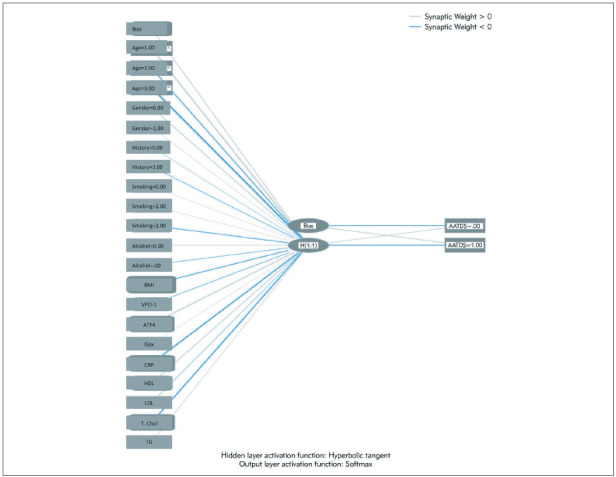
Synaptic weight scheme. »Standardized« method was used for covariates, the number of the hidden layer is »1«, the activation function is »hyperbolic tangent«, and the activation function of the output layer is »sigmoid«.

In Figure 2, we analyzed the synaptic weight of the tested parameters. We used the »standardized« method for covariates; the number of hidden layers was »1«, the activation function was »hyperbolic tangent«, and the activation function of the output layer was »sigmoid«. Our artificial neural networks model, trained using 65 data, has been tested in test data. Estimates were made using our model on 15 patients whose final output was stored. In fact, 11 out of 13 patients who were sick, estimated correctly, so the specificity was found to be 84.6%. The specificity rate for the training data set is 93.6%. Likewise, the sensitivity rate was 50% in test data and training data. The values of the created model were calculated as 81.25%, with 65 correct results for the whole data set. Performance values of our model are over 80% in both data groups. We confirmed the results by ROC graph, where the under curve area (UCA) was 82%.

**Figure 3:**
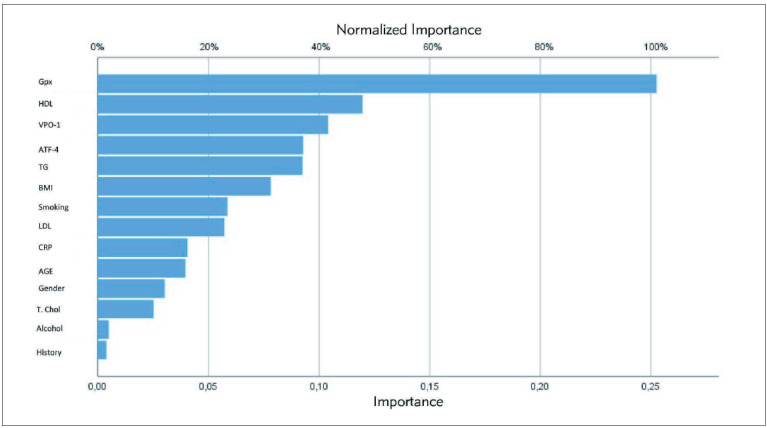
The independent variable importance table shows the importance of the estimating in artificial neural networks algorithm.

The independent variable importance figure shows us which factors are important when estimating artificial neural network algorithm. Accordingly, GPx1, HDL, VPO1 are the most important variables. At the same time, we can say that body mass index (BMI) and smoking history information are more important than age, gender and alcohol consumption parameters in predicting blocked vessels (Figure 3).

## Discussion

Atherosclerosis is a chronic inflammatory disease caused by various factors such as lipids, various genes, immune cells and different kinds of enzymes. Apart from several mechanisms proposed for the pathogenesis of atherosclerosis, there is still a considerable gap in explaining the big picture [12]. In recent studies, the relationship between oxidative stress and endoplasmic reticulum stress became important in the pathogenesis of the disease [13]. Our current study aimed at a preliminary study to determine a biomarker that can contribute to the diagnosis and treatment of coronary artery disease, based on previous research findings in this field.

We observed that VPO1 and CRP levels were significantly higher in single vessel patients than in control subjects, whereas ATF4 and GPx1 levels were lower. In studies examining the relationship between VPO1 and CAD, it is suggested that VPO1 is closely related to oxidative stress and therefore plays an important role in the pathogenesis of the disease [14] [15]. In a rabbit model with myocardial ischemia-reperfusion injury, VPO1 mRNA levels and its expression have been shown to increase with the increase in oxidative damage [16]. Another study conducted on spontaneously hypertensive rabbits revealed that VPO1 expression increased significantly in arterial tissues [17].

In our study, a statistically significant positive correlation was found between VPO1 and ATF4 levels in single-vessel patients. This positive relationship between VPO1 and ATF4 suggests that increased oxidative stress induces the transcription factor ATF4, which actuates the antioxidant defence system. ATF4 is co-activated with UPR in human aortic endothelial cells exposed to oxidized phospholipids, an important trigger of the atherosclerotic process [18]. In studies examining the proapoptotic role of ATF4, it was determined that ATF4 activates the signal pathway in endothelial cells, in which CHOP and ATF3 also take part [19]. On the other hand, ATF4 mediates the upregulation of GRP78, which is one of the major regulators of endoplasmic reticulum stress [20] [21]. ATF4 is thought to protect endothelial cells from oxidative stress-induced cellular damage by these mechanisms and attenuates lipid metabolism [22]. Our study found a statistically significant relationship between ATF4, provoking the antioxidant defence system, triglyceride, total cholesterol, and LDL cholesterol levels, which are substrates of oxidative lipid damage.

In the correlation analysis results in which all our cases were included, it was observed that there was a statistically significant negative relationship between VPO1 and GPx1.

Harding et al. [23] showed that ATF4-mediated integrated stress response provides a supply of amino acids for protein and glutathione biosynthesis and protects cells against oxidative stress. We think that activation of the ATF4-mediated antioxidant system consumes GPx1, a member of this system. GPx1 reduces H_2_O_2_ to water, preventing the formation of more dangerous radical products. Therefore, the decrease in GPx1 levels we detected may actually result from the compensation mechanism for increased H_2_O_2_ clearance. VPO1 uses H_2_O_2_ to form more toxic HOCl in the presence of Cl-ions. Since the substrate of VPO1 will also decrease due to the decreased H_2_O_2_ in the environment, there may be no change in its synthesis [5] [24]. We think that the inverse relationship between GPx1 and VPO1 observed in both the total group and the patient group with a single blocked vessel may be related to the decreased H_2_O_2_ levels.

In our study, a statistically significant negative correlation was found between Gpx1 and CRP levels in two and three-vessel patients. Increasing oxidative stress due to the decrease in Gpx1 levels triggers inflammation. Therefore, increased oxidative stress in the early stages of the disease activates the antioxidant defence system but triggers inflammation in the more advanced stages of the disease [25].

The relationship between oxidative stress and endoplasmic reticulum stress with the pathogenesis of atherosclerosis has not been demonstrated in detail [26]. We believe that our findings will explain the relationships mentioned above and elucidate the pathogenesis of atherosclerosis. In this context, we believe that VPO1 and ATF4 may be biomarkers associated with CAD and will provide significant benefits in the follow-up and monitoring of the treatment process of the disease in addition to traditional risk factors. Our results should be confirmed by extensive studies in different populations and wider patient groups, in which oxidative stress will be evaluated together with protein and lipid damage markers and will be examined with specific vascular inflammation markers.

Azuma et al. [27] developed a good and straightforward statistical model with remarkable accuracy in discriminating coronary artery lesions using common parameters used in clinical medicine. This method helps decide the time and type of therapy for coronary artery disease formation in the future [27]. Kim et al. proposed a new model which may aid in the prevention of heart disease in coronary artery patients. This analysis method might greatly benefit people in terms of predicting, beyond a simple prediction of the coronary artery disease risk and the quantitative survival time [11].

We acknowledge some of the limitations of this study. Patients taking any medication that could affect endothelial function, including statins and antihypertensive agents, were not excluded from the study. Our sample size is small. Female individuals' menopausal status was not evaluated. We defined patients according to their angiography results and evaluated lipid cut-off according to the European Society of Cardiology Score.

## Dodatak

### Acknowledgements

We thank Ozcan Arkan for the assistance of artificial neural network statistical analysis. This work was supported by the grant of the University of Istanbul Research Foundation. The project number is »TYL-2017-22043«.

### Conflict of interest statement

All the authors declare that they have no conflict of interest in this work.

## References

[1] Colles S M, Maxson J M, Carlson S G, Chisolm G M (2001). Oxidized LDL-induced injury and apoptosis in atherosclerosis: Potential roles for oxysterols. Trends Cardiovasc Med.

[2] Libby P, Ridker P M, Maseri A (2002). Inflammation and atherosclerosis. Circulation.

[3] Aladağ N, Asoğlu R, Ozdemir M, Asoğlu E, Derin A R, Demir C, Demir H (2021). Oxidants and antioxidants in myocardial infarction (MI): Investigation of ischemia modified albumin, malondialdehyde, superoxide dismutase and catalase in individuals diagnosed with ST elevated myocardial infarction (STEMI) and non-STEMI (NSTEMI). J Med Biochem.

[4] Levitan I, Volkov S, Subbaiah P V (2010). Oxidized LDL: Diversity, patterns of recognition, and pathophysiology. Antioxid Redox Signal.

[5] Peng H, Zhang K, Liu Z, Xu Q, You B, Li C, Cao J, Zhou H, Li X, Chen J, Cheng G, Shi R, Zhang G (2018). VPO1 modulates vascular smooth muscle cell phenotypic switch by activating extracellular signal-regulated kinase 1/2 (ERK 1/2) in abdominal aortic aneurysms. J Am Heart Assoc.

[6] Nelson R E, Fessler L I, Takagi Y, et al (1994). Peroxidasin: A novel enzyme-matrix protein of drosophila development. EMBO J.

[7] Tang Y, Xu Q, Peng H, et al (2015). The role of vascular peroxidase 1 in ox-LDL-induced vascular smooth muscle cell calcification. Atherosclerosis.

[8] Yang W, Liu Z, Xu Q, et al (2017). Involvement of vascular peroxidase 1 in angiotensin II-induced hypertrophy of H9c2 cells. J Am Soc Hypertens.

[9] Peng H, Chen L, Huang X, Yang T, Yu Z, Cheng G (2016). Vascular peroxidase 1 up regulation by angiotensin II attenuates nitric oxide production through increasing asymmetrical dimethylarginine in HUVECs. J Am Soc Hypertens.

[10] Liu W Q, Zhang Y Z, Wu Y, Zhang J Z, Li T, Jiang T, Xiong X, Luo X, Ma Q, Peng J (2015). Myeloperoxidase-derived hypochlorous acid promotes ox-LDL-induced senescence of endothelial cells through a mechanism involving b-catenin signaling in hyperlipidemia. Biochem Biophys Res Commun.

[11] Kim J K, Kang S (2017). Neural network-based coronary heart disease risk prediction using feature correlation analysis. J Healthc Eng.

[12] Andreou D E, Andreadou I (2009). Atherosclerosis: An inflammatory disease. Pharmakeftiki.

[13] Zeeshan H M A, Lee G H, Kim H R, Chae H J (2016). Endoplasmic reticulum stress and associated ROS. Int J Mol Sci.

[14] Ma Q L, Zhang G G, Peng J (2013). Vascular peroxidase 1: A novel enzyme in promoting oxidative stress in cardiovascular system. Trends Cardiovasc Med.

[15] Li T, Zhang Y, He L, Liu B, Shi R, Zhang G, Peng J (2012). Inhibition of vascular peroxidase alleviates cardiac dysfunction and apoptosis induced by ischemia-reperfusion. Can J Physiol Pharmacol.

[16] Zhang Y Z, Wang L, Zhang J J, Xiong X M, Zhang D, Tang X M, Luo X J, Ma Q L, Peng J (2018). Vascular peroxide 1 promotes ox-LDL-induced programmed necrosis in endothelial cells through a mechanism involving β-catenin signaling. Atherosclerosis.

[17] Shi R, Hu C, Yuan Q, et al (2011). Involvement of vascular peroxidase 1 in angiotensin II-induced vascular smooth muscle cell proliferation. Cardiovasc Res.

[18] Gargalovic P S, Imura M, Zhang B, Gharavi N M, Clark M J, Pagnon J, Yang W, He A, Truong A, Patel S, Nelson S F, Horvath S, Berliner J A, Kirchgessner T G, Lusis A J (2006). Identification of inflammatory gene modules based on variations of human endothelial cell responses to oxidized lipids. Proc Natl Acad Sci U S A.

[19] Mungrue I N, Pagnon J, Kohannim O, Gargalovic P S, Lusis A J (2009). CHAC1/MGC4504 is a novel proapoptotic component of the unfolded protein response, downstream of the ATF4-ATF3-CHOP cascade. J Immunol.

[20] Wu H L L, Li Y H H, Lin Y H H, Wang R, Li Y B H, Tie L, Song Q L, Guo D A, Yu H M, Li X J H (2009). Salvianolic acid B protects human endothelial cells from oxidative stress damage: A possible protective role of glucose-regulated protein 78 induction. Cardiovasc Res.

[21] Fusakio M E, Willy J A, Wang Y, Mirek E T, Baghdadi A R J T, Adams C M, Anthony T G, Wek R C (2016). Transcription factor ATF4 directs basal and stress-induced gene expression in the unfolded protein response and cholesterol metabolism in the liver. Mol Biol Cell.

[22] Wang C, Huang Z, Du Y, Cheng Y, Chen S, Guo F (2010). ATF4 regulates lipid metabolism and thermogenesis. Cell Res.

[23] Harding H P, Zhang Y, Zeng H, Novoa I, Lu P D, Calfon M, Sadri N, Yun C, Popko B, Paules R, Stojdl D F, Bell J C, Hettmann T, Leiden J M, Ron D (2003). An integrated stress response regulates amino acid metabolism and resistance to oxidative stress. Mol Cell.

[24] Zhang Y S, He L, Liu B, Li N, Luo X, Hu C, Ma Q, Zhang G S, Li Y, Peng J (2012). A novel pathway of NADPH oxidase/vascular peroxidase 1 in mediating oxidative injury following ischemia-reperfusion. Basic Res Cardiol.

[25] Libby P, Tabas I, Fredman G, Fisher E A (2014). Inflammation and its resolution as determinants of acute coronary syndromes. Circ Res.

[26] Reiner Z E, Catapano A L, Backer G D, Graham I, Taskinen M R, Wiklund O, Agewall S, Alegria E, Chapman M J, Durrington P, Erdine S, Halcox J, Hobbs R, Kjekshus J, Filardi P P, Riccardi G, Storey R F, Wood D (2011). ESC/EAS guidelines for the management of dyslipidaemias: The task force for the management of dyslipidaemias of the European Society of Cardiology (ESC) and the European Atherosclerosis Society (EAS). Eur Heart J.

[27] Azuma J, Yamamoto T, Nitta M, Hasegawa Y, Kijima E, Shimotsuji T, Mizoguchi Y (2020). Structure equation model and neural network analyses to predict coronary artery lesions in Kawasaki disease: A single-centre retrospective study. Sci Rep.

